# Fabrication of biphasic cartilage-bone integrated scaffolds based on tissue-specific photo-crosslinkable acellular matrix hydrogels

**DOI:** 10.1016/j.mtbio.2022.100489

**Published:** 2022-11-08

**Authors:** Yujie Hua, Yingying Huo, Baoshuai Bai, Junxiang Hao, Guanhuai Hu, Zheng Ci, Xiaodi Wu, Mengyuan Yu, Xin Wang, Hong Chen, Wenjie Ren, Yixin Zhang, Xiaoyun Wang, Guangdong Zhou

**Affiliations:** a Department of Plastic and Reconstructive Surgery, Shanghai Ninth People's Hospital, Shanghai Jiao Tong University School of Medicine, Shanghai Key Laboratory of Tissue Engineering, Shanghai, PR China; b Department of Plastic Surgery, Tongren Hospital, Shanghai Jiao Tong University School of Medicine, Shanghai Key Laboratory of Tissue Engineering, Shanghai, PR China; c Research Institute of Plastic Surgery, Weifang Medical University, Weifang, Shandong, PR China; d National Tissue Engineering Center of China, Shanghai, PR China; e Institute of Regenerative Medicine and Orthopedics, Institutes of Health Central Plain, Xinxiang Medical University, Xinxiang, Henan, PR China; f Department of Orthopaedics, Qilu Hospital of Shangdong University Centre for Orthopaedics, Advanced Medical Research Institute, Shangdong University, Shangdong, PR China; g Department of Hand Surgery, Ningbo Sixth Hospital, Zhejiang, PR China

**Keywords:** Photo-crosslinkable hydrogels, Tissue-specific acellular matrix, Biphasic cartilage-bone integrated scaffolds, Osteochondral repair)

## Abstract

The fabrication of biphasic cartilage-bone integrated scaffolds is an attractive alternative for osteochondral repair but has proven to be extremely challenging. Existing three-dimensional (3D) scaffolds are insufficient to accurately biomimic the biphasic cartilage-bone integrated microenvironment. Currently, photo-crosslinkable hydrogels based on tissue-specific decellularized extracellular matrix (dECM) have been considered as an important technique to fabricate biomimetic scaffolds, but so far there has been no breakthrough in the photo-crosslinkable hydrogel scaffolds with biphasic cartilage-bone biomimetic microenvironment. Here, we report a novel strategy for the preparation of biomimetic cartilage-bone integrated scaffolds based on photo-crosslinkable cartilage/bone-derived dECM hydrogels, which are able to reconstruct biphasic cartilage-bone biomimetic microenvironment. The biphasic cartilage-bone integrated scaffolds provided a 3D microenvironment for osteochondral regeneration. The cartilage biomimetic scaffolds, consisting of cartilage-derived dECM hydrogels, efficiently regulated chondrogenesis of bone marrow mesenchymal stem cells (BMSCs). The bone biomimetic scaffolds, composed of cartilage/bone-derived dECM hydrogels, first regulated chondrogenesis of BMSCs, followed by endochondral ossification over time. Taken together, the biphasic cartilage-bone integrated tissue could be successfully reconstructed by subcutaneous culture based on cartilage-bone bilayered structural design. Furthermore, the biphasic cartilage-bone biomimetic scaffolds (cell-free) achieved satisfactory cartilage-bone integrated regeneration in the osteochondral defects of rabbits’ knee joints.

## 1 Introduction

Articular cartilage defect is usually accompanied by subchondral bone degeneration, which is an important and intractable clinical problem [[1], [2], [3], [4], [5], [6], [7]]. At present, tissue engineering is emerging as a promising alternative approach: seeding cells on biomaterials to reconstruct desired substitutes for tissue repair [[8], [9], [10], [11], [12]]. Although some strategies for cartilage or bone regeneration based on bone marrow mesenchymal stem cells (BMSCs) have been established [[13], [14], [15], [16], [17], [18], [19], [20]], a viable strategy for fabricating cartilage-bone integrated tissue has not yet developed. This is due to the lack of ideal biphasic cartilage-bone integrated scaffolds with accurate biomimic of the tissue-specific microenvironment, which has proven to be extremely challenging to reconstruct.

Currently, photo-crosslinkable hydrogels based on tissue-specific decellularized extracellular matrix (dECM) have been considered one of the most suitable choices to fabricate biomimetic scaffolds, because of the spatiotemporal controllability of the photo-crosslinking method [[21], [22], [23], [24], [25], [26]] and the accurate microenvironment biomimic of tissue-specific dECM with respect to intricate composition, architecture, and topological structure [[27], [28], [29], [30], [31]]. To date, photo-crosslinkable dECM-derived scaffolds have mainly focused on soft tissue and organs, such as kidney or muscle [30,31], and few studies have reported constructing dECM-derived photo-crosslinkable hydrogels based on hard tissue, such as cartilage or bone [[32], [33], [34], [35]]. Due to the compact structure of cartilage and highly mineralized components of bone [[36], [37], [38]], they are both quite difficult to prepare as soluble photo-crosslinkable dECM hydrogel. To address this problem, complex treatments by physical, chemical, or enzymatic approaches are essential, which led to poor photo-crosslinking properties and partial loss of tissue-specific biomemitic ingredients. Moreover, based on the principle of developmental biology, the regeneration of subchondral bone in joint is dominantly achieved by the endochondral ossification pathway [[39], [40], [41]]. Thus, an ideal biphasic cartilage-bone integrated scaffold would go beyond a standard 3D culture model to create a physiologically biomimetic tissue regeneration system [[42], [43], [44]], which can better mimic the time-dependent physiological process of endochondral ossification (the fourth dimension). Because of the above difficulties and complicated requirements, biphasic cartilage-bone integrated scaffolds with accurate tissue-specific biomimetic microenvironments and dynamic regulation of endochondral ossification have not yet been developed. Collectively, the development of the biphasic cartilage-bone integrated scaffold needs to solve the following problems: 1) how to prepare cartilage- and bone-specific photo-crosslinkable hydrogels with tissue-specific biomemitic ingredients and satisfactory gelling properties; 2) whether the cartilage biomimetic scaffolds could regulate chondrogenesis of BMSCs; 3) whether the bone biomimetic scaffolds could regulate osteogenesis of BMSCs by time-dependent endochondral ossification; 4) whether biphasic cartilage-bone integrated tissue could be successfully regenerated with satisfactory interfacial integration based on the biphasic biomimetic scaffolds; 5) whether articular osteochondral defects could be *in situ* repaired with regenerated cartilage and subchondral bone based on the cell-free biphasic biomimetic scaffolds.

To address the above issues, we here proposed a novel strategy for the development of biphasic cartilage-bone integrated scaffolds based on cartilage/bone-derived photo-crosslinkable dECM (**photo-dECM**) hydrogels, which were able to achieve biomimic and reconstruction of the biphasic and dynamic cartilage-bone microenvironment. The biphasic scaffold contained a biomimetic cartilage microenvironment, comprising photo-crosslinkable cartilage-derived dECM (**photo-dECM**_**C**_) hydrogels, and a biomimetic bone microenvironment, comprising a combination of **photo-dECM**_**C**_ hydrogels and decalcified bone matrix (**DBM**). This provided both chondrogenic and osteogenic microenvironments for the time-dependent endochondral ossification pathway. The feasibility of cartilage, bone, and biphasic cartilage-bone integrated tissue regeneration were investigated using tissue-specific biomimetic scaffolds combined with chondrocytes or BMSCs. Finally, osteochondral defect repair in rabbits’ knee joints was further evaluated by *in situ* gelling of the cell-free biphasic cartilage-bone integrated scaffolds.

## 2 Materials and methods

### 2.1 Materials and animals

In this study, trypsin, collagenase, pepsin, Triton X-100, methacrylic anhydride, sodium hydroxide, lithium phenyl-2,4,6-trimethylbenzoylphosphinate (LAP), and 4-(4,6-dimethoxy-1,3,5-triazin-2-yl)-4-methyl morpholinium chloride were purchased from Sigma-Aldrich. All other chemicals were reagent grade and deionized water was used. Both nude mice and New Zealand white rabbits were purchased from Shanghai Jiagan Experimental Animal Raising Farm (Shanghai, China). All protocols for animal experiments were approved by the Animal Care and Experimental Committee of Shanghai Jiao Tong University School of Medicine (SH9H-2021-A655-SB).

### 2.2 Preparation of photocrosslinkable dECM

Total cartilage (derived from ear, joint, and meniscus) and skin tissue were obtained from pigs that were purchased from a local slaughterhouse. Photo-crosslinkable dECM polymers were synthesized with the following three steps: 1) Powder-like dECM preparation: Fresh tissue was first ground into powder by freezer mixer, and treated with 0.5% trypsin/phosphate-buffered saline (PBS) (w/v) for 24 ​h. Samples were then sequentially treated with nuclease solution (containing 50 U/ml deoxyribonuclease and 1 U/ml ribonuclease A in 10 ​mM Tris-HCL, pH ​= ​7.5) for 4 ​h, 10 ​mM Tris-HCL (including 10 U/ml aprotinin) for 20 ​h, and 1% Triton X-100/PBS solution (v/v) for 24 ​h. Powder-like dECM was prepared by freeze-drying after washing six times in PBS for 8 ​h. Morphology of dECM powder was observed using light microscopy (Olympus CKX41) and scanning electron microscopy (SEM; Philips XL-30, Amsterdam, Netherlands) at an accelerating voltage of 10 ​kV. 2) Water-soluble treatment of dECM: Cartilage-derived dECM was treated with 0.15% collagenase, and skin-derived collagen was treated with 0.2% pepsin (0.1 ​M HCL solution) for 24 ​h. The solution was dialyzed against deionized water for 3 days, followed by freezing and lyophilizing. 3) Methacrylation of dECM: The methacrylation process was performed as previously reported [36]. Briefly, 0.5 ​g water-soluble dECM or collagen was dissolved in deionized water, and methacrylic anhydride (0.5 ​mL) was added drop wise in an ice bath. The pH was maintained between 8 and 11 with adjustment by 5 ​M NaOH (aq) and the reaction continued overnight in the dark at 4 ​°C. After the reaction, the solution was centrifuged to remove insoluble substances, and the pH was adjusted to 7.4 by 1 ​M HCl (aq). Crude product was dialyzed against deionized water for 3 days, followed by freezing and lyophilizing. ^1^H NMR analysis was performed to determine the degree of methacrylation as previously described [46]. Hydrogel precursors of dECM-MA, ColMA, and LAP (0.2% w/v) were mixed according to different requirements in D-PBS (pH 7.4) at 37 ​°C. Then, the above specimens were subjected to different measurements following light irradiation (365-nm LED, 20 ​mW/cm^2^).

### 2.3 Component analysis of dECM

Quantification of DNA content was performed using a Quant-IT PicoGreen dsDNA Assay Kit 2000 (Life Technologies, Carlsbad, CA, USA) according to the manufacturer's instructions. Glycosaminoglycan (GAG) content was analyzed by the spectrophotometric microdetermination dimethylmethylene blue assay (DMMB, Sigma-Aldrich), and total collagen content was quantified by hydroxyproline assay kit (Sigma-Aldrich). The possible collagen contents in dECM_C_ and dECM_B_ were analyzed by proteomics, and Kyoto Encyclopedia of Genes and Genomes (KEGG) pathway mapping was applied to infer the potential mechanism of materials' function.

### 2.4 Rheological analysis of photo-dECM

Dynamic rheology experiments were performed on a HAAKE MARS Ⅲ photorheometer with parallel-plate (P20 TiL, 20-mm diameter) geometry and OmniCure Series 2000 (365 ​nm, 20 ​mW/cm^2^) at 25 ​°C. Time sweep oscillatory tests were performed at a 10% strain (CD mode), 1 ​Hz frequency and a 0.5 ​mm gap for 180 ​s. Strain sweeps were performed to verify the linear response. The gel point was determined as the time when the storage modulus (G′) surpassed the loss modulus (G″). The elastic modulus was determined as the storage modulus (G’) reaching to the complete gelation.

### 2.5 Enzyme-mediated degradation test

Weight of the formed photo-dECM hydrogel was recorded as W_0_, then the gel was incubated in D-PBS (pH ​= ​7.4) supplemented with hyaluronidase solution (50 U/mL in D-PBS), or collagenase solution (50 U/mL in D-PBS). At each time point, these samples were collected, gently blotted with filter paper to get rid of excess water on the surface, and recorded as the weight W_t_. The mass loss (%) was calculated according to the following equation:

$$Mass\;loss\;\left(\%\right)=\frac{W_{o}-W_{t}}{W_{o}}\times 100\%$$

### 2.6 Cell isolation and culture

For chondrocyte culture, fresh auricular cartilage was obtained from New Zealand rabbit ear and minced into small pieces. The cartilage pieces were digested using 0.15 ​wt% type II collagenase (Worthington Biochemical Corp., Freehold, NJ, USA) to isolate chondrocytes. The cells were harvested, cultured, and expanded in culture medium (high glucose Dulbecco's Modified Eagle Medium [DMEM, Gibco BRL, Grand Island, NY, USA], 10% fetal bovine serum [FBS, Hyclone, Logan, UT, USA], and 1% penicillin/streptomycin/amphotericin B solution) supplemented with 5 ​ng/mL basic fibroblast growth factor (bFGF) [47,48]. The P_0_ chondrocytes were passaged at the ratio of 1:5. Then, chondrocytes in the second passage were harvested for cartilage regeneration.

For BMSC culture, fresh bone marrow was extracted from New Zealand rabbits, and flushed out into culture dishes with stem cell culture medium (MesenCult™ MSC Basal Medium). Non-adherent cells were discarded by changing the culture medium after 5d culture. The P_0_ BMSCs were passaged at the ratio of 1:5. BMSCs in the second passage were harvested for bone regeneration.

### 2.7 Cytocompatibility evaluation

Cytotoxicity was assessed using the CCK-8 method. Briefly, chondrocytes or BMSCs were seeded in 96-well plates at a concentration of 5000 ​cells per well and cultured for 24 ​h. The photo-dECM polymers (0.5% w/v) were dissolved in D-PBS and sterilized using a filtration membrane (0.22 ​μm). The culture solution was removed after 1, 4, and 7 ​d of incubation, and 10 ​μL CCK-8 reagent and 100 ​μL culture medium were added to each well and incubated for 2 ​h at 37 ​°C. The absorbance was measured using a microplate reader (Synergy H1, BioTek) at 450 ​nm.

### 2.8 In vitro chondrogenic and osteogenic experiments

BMSCs were seeded in 96-well plates at a concentration of 1 ​× ​10^5^ ​cells per well and cultured for 24 ​h. Gel precursors (**dECM**_**C**_, **dECM**_**S**_, **GelMA**, and **HAMA**, 1% w/v) or powders of decalcified bone matrix (0.5% w/v) in culture medium [low-glucose Dulbecco's modified Eagle medium (Gibco BRL, Grand Island, NY), 10% fetal bovine serum (Hyclone, Logan, UT, USA), and 1% penicillin/streptomycin/amphotericin B solution] were used to culture BMSCs *in vitro*. The new culture medium with additive gel precursors were changed every three days. After 7 or 14 days of growth, cells were collected for chondrogenic staining (F-actin, Alcian blue, and COL2), or osteogenic staining (F-actin, ALP, and COL1). For hydrostatic bioreactor culture *in vitro*, BMSC-loaded photo-dECM hydrogels were applied upon hydrostatic pressure stimulation at 5 ​MPa for 2 ​h once a day.

### 2.9 Quantitative real-time PCR (qRT-PCR) and western blot (WB) analysis

Total RNA from BMSCs of chondrogenic and osteogenic stimulation and engineered cartilage or bone were extracted and reverse-transcribed using a PrimeScript RT reagent Kit (TaKaRa, Japan) according to the manufacturer's protocol. Gene expression was quantified from cDNA with qRT-PCR using SYBR Premix EX Taq (TaKaRa, Japan). The relative expression was calculated using the 2^−ΔCT^ method for the following genes: cartilage-related genes (*COL2*, *AGG*, and *SOX9*), and bone-related genes (*COL1*, *ALP*, *OCN* and *RUNX2*). Total chondrogenic and osteogenic proteins were extracted using a protein extraction kit (Biorab, China). Protein samples were resolved by electrophoresis and were transferred to polyvinylidene fluoride membranes. After blocking, membranes were incubated with specific primary antibodies for cartilage-related proteins (COL2, and AGG), bone-related proteins (COL1, and ALP) and GAPDH overnight at 4 ​°C, followed by an incubation with a secondary antibody for 1 ​h at room temperature. The immunoreactive protein bands were detected using an electrochemiluminescene (ECL) reagent (Millipore, USA).

### 2.10 In vivo cartilage and bone regeneration

Chondrocytes or BMSCs were isolated, cultured, and expanded as the above methods. The cells (Passage 2) were resuspended in the gel precursor solution for the preparation of cell-loaded hydrogels. Then, the gel precursor solution loaded with 50 ​× ​10^6^ ​mL^−1^ chondrocytes or 20 ​× ​10^6^ ​mL^−1^ BMSCs were poured into cylindrical models (10 ​mm in diameter and 2 ​mm in height) to fabricate cell-loaded hydrogels upon light irradiation (365 ​nm LED, 20 ​mW/cm^2^). The cell-loaded hydrogels were then subcutaneously implanted into nude mice. At 4 or 8 weeks after implantation, all samples were harvested to evaluate cartilage or bone regeneration. The hematoxylin and eosin (H&E), Safranin-O, Alcian blue, and type Ⅱ collagen staining were used to evaluate the cartilage-specific ECM deposition, while hematoxylin and eosin (H&E), Safranin-O/fast green, and Masson's trichrome staining were used to evaluate the bone-specific ECM deposition.

### 2.11 Micro computed tomography (micro-CT) analysis

At 4 and 8 weeks post-implantation, the new engineered bone was assessed using a micro-CT system (SkyScan 1172, Bruker, USA) with the following parameters: resolution of the scans was 20 ​μm, source voltage was 100 ​kV. The morphometric indices included bone volume (BV) and bone volume fraction (bone volume/tissue volume ratio, BV/TV).

### 2.12 In vivo cartilage-bone integrated tissue regeneration

The gel precursor solution loaded with 50 ​× ​10^6^ ​mL^−1^ chondrocytes and 20 ​× ​10^6^ ​mL^−1^ BMSCs were poured into cylindrical models (10 ​mm in diameter and 4 ​mm in height) in sequence to fabricate the bilayered structure (1 ​mm in height cartilage layer and 3 ​mm in height bone layer) upon light irradiation (365 ​nm LED, 20 ​mW/cm^2^). Cell-loaded hydrogels were then subcutaneously implanted into nude mice. At 4 and 8 weeks post-implantation, all samples were harvested to evaluate cartilage-bone integrated tissue regeneration. The histological examination and qPCR analysis were described above.

### 2.13 Repair of rabbit osteochondral defect

A rabbit osteochondral defect model was created to evaluate the repair effect of the biomimetic cartilage-bone integrated scaffold *in vivo*. Briefly, a total of 8 New Zealand white rabbits were randomly divided into two groups: hydrogel-treated group (Exp group) and non-treated group (Ctrl group). An osteochondral defect (a cylinder 4 ​mm in diameter and 4 ​mm in depth) was created using a sterile drill in the center of the trochlear groove on one knee of the rabbit. Subsequently, the gel precursor of photo-dECM_C_/Col/dECM_B_ was firstly injected into the target defect site and immediately irradiated for 30 ​s (365 ​nm, 20 ​mW/cm^2^) to construct bone phasic hydrogels, and followed by the formation of photo-dECM_C_/Col in the same way to construct cartilage phasic hydrogels, while the defects in the Ctrl group were left untreated.

### 2.14 Gross observation, histological examination, and qPCR analysis of repaired tissue

Three months after the operation, all rabbits were euthanized to harvest repaired knee joints. Harvested tissues were sawed sagittally at the midline of repaired regions for gross observation, and histological examination. All the samples were fixed in 4% paraformaldehyde, decalcified in 15% (w/v) ethylenediamine-tetra acetic acid (EDTA), dehydrated through an ethanol series, embedded in paraffin, and sectioned for histological analysis. Sections were stained with hematoxylin and eosin (H&E), Safranin-O, and Masson's trichrome staining to evaluate histological structure and cartilage- and bone-specific ECM deposition in the repaired regions. The histological scores of the osteochondral joints at 12 weeks were referred to the previous report [49]. The chondrogenic expression levels (*COL2*, *AGG*, and *SOX9*) of the cartilage layer and the osteogenic expression levels (*COL1*, *ALP*, *OCN* and *RUNX2*) of the bone layer were also analyzed by qPCR as described above.

### 2.15 Statistical analysis

All data are presented as mean ​± ​standard deviation (s.-d.). Differences between the values were evaluated using one-way analysis of variance (ANOVA) with p ​< ​0.05 considered statistically significant.

## 3 Results

### 3.1 Preparation and characterization of photo-dECM hydrogels

**Fig. 1:**
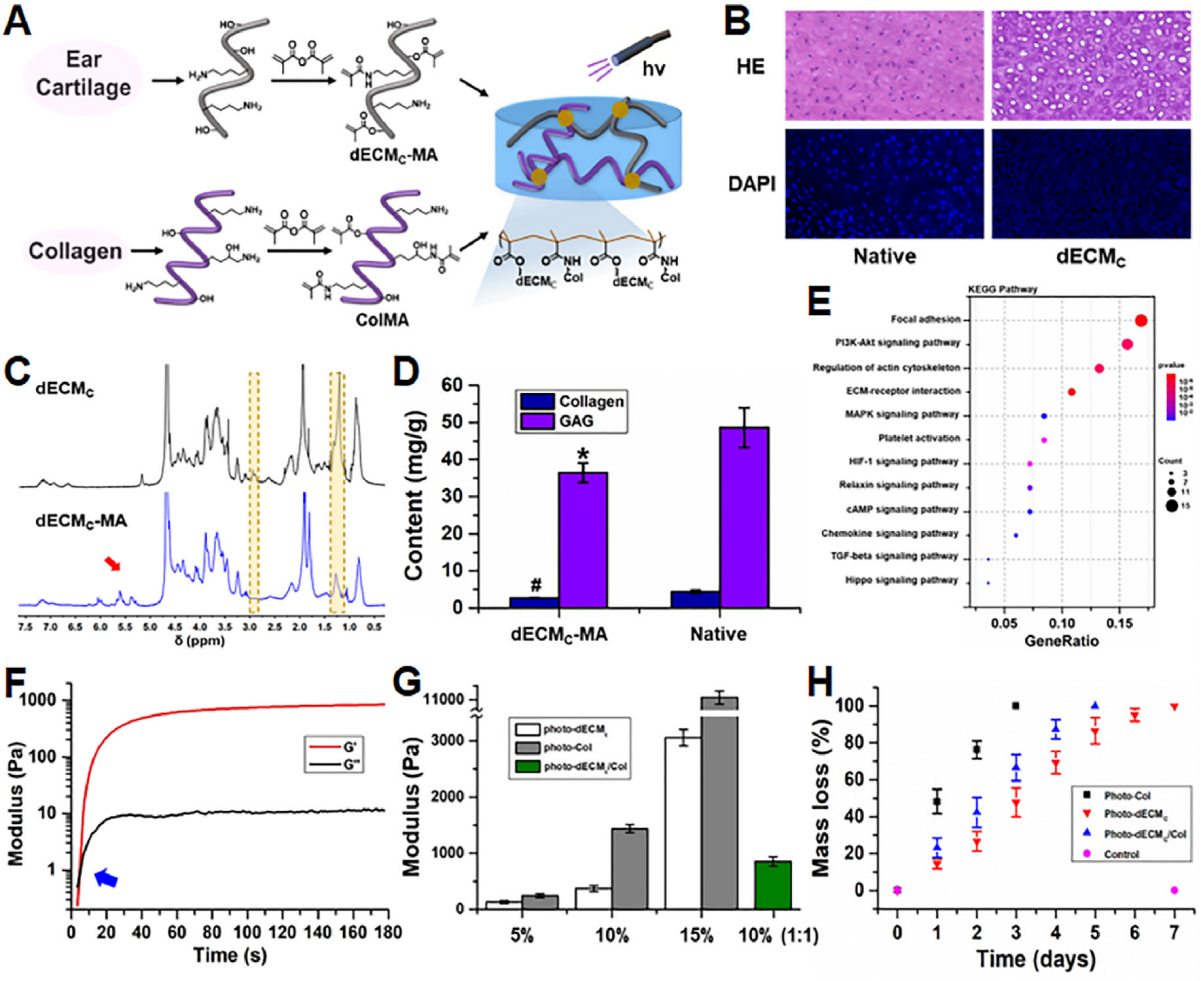
Characterization of photo-dECM hydrogel. (A) Schematic illustration of the fabrication of photo-crosslinkable dECM hydrogel. (B) Histological images of H&E, and DAPI staining of ear cartilage tissue before and after decellularization. (C) ^1^H NMR spectra of **dECM**_**C**_ and **dECM**_**C**_**-MA** polymers. (D) Glycosaminoglycan (GAG) and collagen contents before and after decellularization (^#^p, ∗p ​< ​0.05, compared with the native tissue). (E) The statistical analysis of KEEG pathway of **dECM**_**C**_. (F) Rheological analysis of **photo-dECM**_**C**_**/Col** hydrogel (10% w/v, **dECM**_**C**_**-MA**:**ColMA** ​= ​1:1). (G) Comparison of elastic modulus with different solid contents. (H) Hydrogels degraded in response to enzyme solution, such as **photo-dECM**_**C**_ hydrogel in hyaluronidase solution (50 U/mL in D-PBS), **ColMA** hydrogel in collagenase solution (50 U/mL in D-PBS), and **photo-dECM**_**C**_**/Col** hydrogel in mixed enzyme solution (50 U/mL hyaluronidase and collagenase in D-PBS).

In this study, the photo-crosslinkable dECM-based hydrogels were synthesized with the following three steps: 1) preparation of powder-like dECM through the decellularization procedure [36]; 2) water-soluble treatment of dECM by enzymatic methods, *e.g.*, collagenase, or pepsin; 3) methacrylation of dECM to synthesize the methacrylate-modified dECM (**dECM-MA**), which is the common methacrylation reaction to prepare the photo-crosslinkable hydrogel, *e.g.*, GelMA [45], HAMA [46], and muscle-dECM based hydrogel [31]. Porcine cartilage tissues were used to prepare cartilage-derived photo-dECM, and skin-derived collagen was extracted to prepare photo-crosslinkable collagen (**photo-Col**) to fabricate hybrid hydrogels (Fig. 1A and B and Figs. S1–2). ^1^H NMR spectroscopy of the **dECM-MA** polymers showed signal peaks of 5.2–6.2 ​ppm, indicating the successful graft of methacrylate groups (Fig. 1C and Fig. S3). The substitution degree, determined by the integral ratio of the proton peaks at 5.2–6.2 ​ppm to the peak at 2.9 and 1.3 ​ppm (amino and hydroxyl groups of dECM), indicated 87% of methacrylate-modified dECM_C_ (**dECM**_**C**_**-MA**), and 91% of methacrylate-modified collagen (**ColMA**). Tissue decellularization was confirmed by histological examination and testing relative DNA contents (Fig. 1B and S2a). Additionally, glycosaminoglycan (GAG) and collagen contents showed that a majority of GAG components remained in the **dECM**_**C**_**-MA** after collagenase treatment, while most of the collagen components were lost (Fig. 1D). The combination of **dECM**_**C**_**-MA** with **ColMA** (**photo-dECM**_**C**_**/Col** hydrogel) can supplement enough collagen components to reconstruct ECM that containing both glycosaminoglycans and glycoproteins. Moreover, proteomic analysis displayed the existence of corresponding cartilage-specific pathway factors in the **dECM**_**C**_ components (Fig. 1E).

Next, the rheological properties of **photo-dECM** hydrogels were measured by *in situ* photo-rheometer. We found that the **photo-dECM**_**C**_**/Col** hydrogels gelled in approximately 5 ​s through photo-initiated radical polymerization reaction (Fig. 1F). The elastic modulus could be varied from ∼200 ​Pa to ∼12000 ​Pa by adjusting solid contents (Fig. 1G). Notably, the multiple components mixed with **dECM**_**C**_**-MA** and **ColMA** could better balance the hydrogel ingredients containing both GAGs and collagen with satisfactory elastic modulus (∼800 ​Pa). We further investigated the gelling properties of **dECM-MA** derived from different tissues, such as ear, joint, and meniscus, which exhibited extensive photo-crosslinkable universality with reasonable mechanical properties (Fig. S4). Furthermore, the degradation properties of **photo-dECM** hydrogels demonstrated that the **photo-dECM**_**C**_ hydrogel was degraded in the hyaluronidase solution (50 U/mL in D-PBS) within 7 ​d, while **ColMA** hydrogel was degraded in the collagenase solution (50 U/mL in D-PBS) in less than 3 ​d (Fig. 1H). The **photo-dECM**_**C**_**/Col** hydrogel displayed a gradually degradable profile in a mixed enzyme solution (50 U/mL hyaluronidase and collagenase in D-PBS).

### 3.2 Chondrogenesis evaluation and engineered cartilage regeneration

**Fig. 2:**
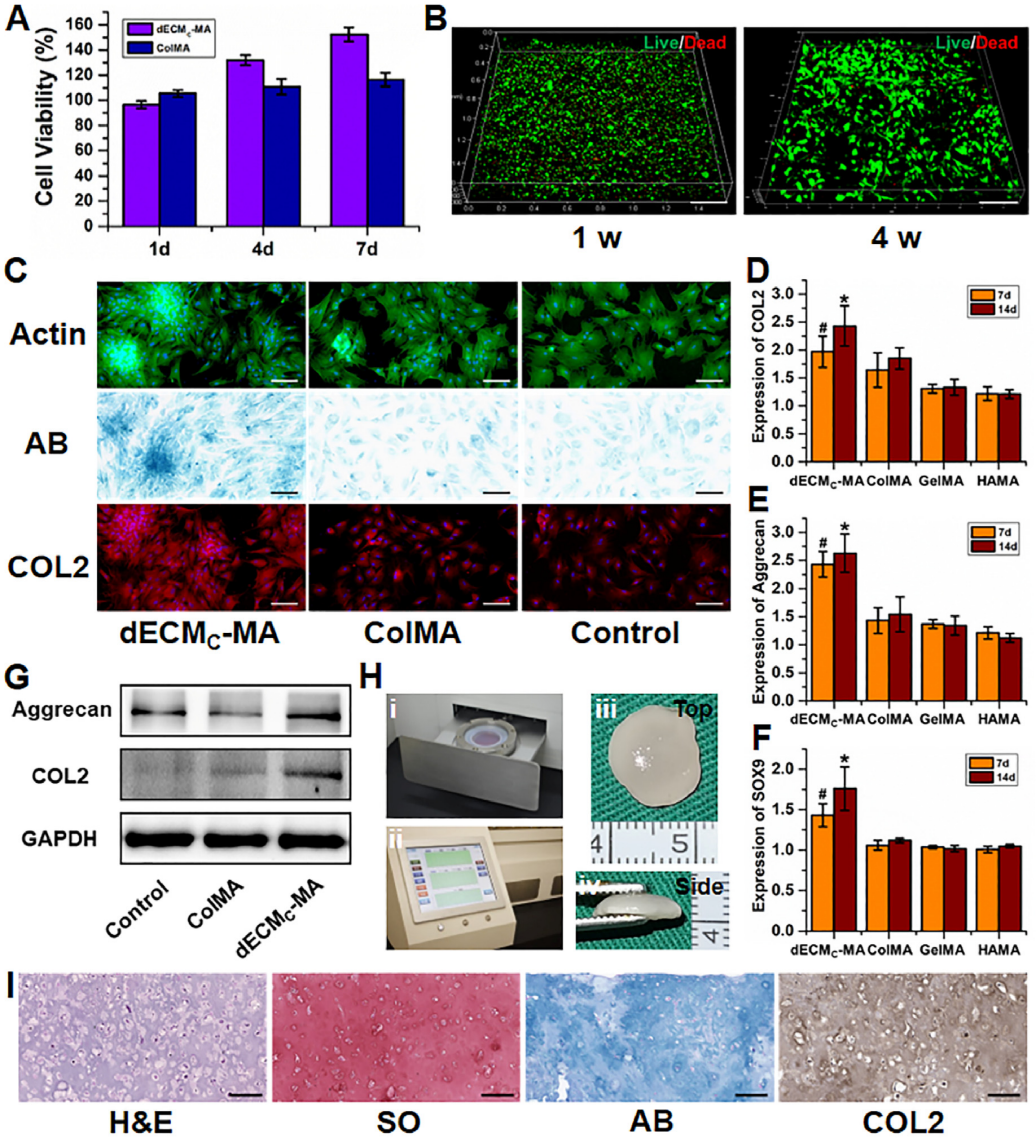
*In vitro* chondrogenesis evaluation and engineered cartilage regeneration. (A) Cell viability of BMSCs in the gel precursor of **photo-dECM** hydrogels evaluated by CCK-8 assay (n ​= ​4). (B) Live/dead staining (live: green; dead: red) of the BMSCs *in situ* encapsulated in **photo-dECM** hydrogels after cultivating for 1 and 4 weeks. (C) Chondrogenic staining of F-actin, Alcian Blue (AB), and COL2 of BMSCs co-cultured with or without gel precursor (**dECM**_**C**_**-MA** and **ColMA**) at day 14. (D–F) Expression of chondrogenic genes (*COL2*, *AGG*, and *SOX9*) in BMSCs cultured with gel precursor (**dECM**_**C**_**-MA**, **ColMA**, **GelMA**, and **HAMA**, 1% w/v) at day 7 and 14 (^#^p, ∗p ​< ​0.01, compared with the **ColMA** group at the same time point). (G) Western blot analysis of aggrecan, COL2 and GAPDH of BMSCs co-cultured with or without gel precursor (**dECM**_**C**_**-MA** and **ColMA**) at day 14. (H) Photographs of hydrostatic bioreactor of culture tank (i) and mechanical control system (ii). Gross view of the regenerated cartilage cultured by BMSC-loaded **photo-dECM** hydrogels at 8 weeks *in vitro* (iii, iv). (I) Representative histological images of H&E, Safranin-O, Alcian Blue, and type Ⅱ collagen staining of the regenerated cartilage cultured by BMSC-loaded **photo-dECM** hydrogels at 8 weeks *in vitro*. Scale bar ​= ​200 ​μm.

Before investigating the cartilage regenerative capability, cytocompatibility was evaluated using cell viability assays and live/dead staining. The current results showed that the dECM gel precursor did not show obvious cytotoxicity (>94% cell viability) for either chondrocytes or BMSCs. Importantly, compared to the **ColMA** polymer, cartilage-derived dECM promoted more cell proliferation due to the presence of potential bioactive ingredients in **dECM**_**C**_**-MA** (Fig. 2A and S5). Live/dead staining demonstrated that BMSCs encapsulated in the **photo-dECM** hydrogel (10% w/v **photo-dECM**_**C**_**/Col**; **dECM**_**C**_**-MA**:**ColMA** ​= ​1:1) showed obvious proliferation and spreading within the hydrogels after 4 weeks of culture, indicating that **photo-dECM**_**C**_**/Col** hydrogel showed satisfactory cytocompatibility and favorable attributes as a tissue-engineered scaffold (Fig. 2B).

The chondrogenic potential of cartilage-derived dECM was further investigated by co-culture with chondrocytes or BMSCs. As shown in Fig. 2C and S6, both chondrocytes and BMSCs co-cultured with cartilage-derived dECM (**dECM**_**C**_**-MA** group) displayed distinct cell aggregation behavior with more GAG and type II collagen deposition (supported by Alcian Blue and collagen II staining) compared to **ColMA** and non-treated (control) groups. Furthermore, expression of both cartilage-specific genes (*COL2*, *AGG*, and *SOX9*) and proteins (COL2 and AGG) were significantly upregulated in the **dECM**_**C**_**-MA** group compared to the **ColMA** group and to common photo-sensitive polymer (such as GelMA or HAMA), indicating that cartilage-derived dECM could effectively promote chondrogenesis (Fig. 2D–G).

Cartilage formation is the most important criterion for determining whether the **photo-dECM** scaffold is useful for cartilage engineering. *In vivo* results demonstrated that chondrocyte-loaded **photo-dECM** hydrogels successfully served as cartilage-regenerating scaffolds, regenerating mature cartilage tissue in the subcutaneous environment of nude mice after 8 weeks of culture (Fig. S7). *In vitro* results demonstrated that BMSC-loaded **photo-dECM** hydrogels cultured in a hydrostatic bioreactor could successfully regenerate cartilage-like tissue with cartilage-specific ECM deposition and typical lacuna structure (Fig. 2H and I). Quantitative analysis further supported *in vitro* cartilage regeneration of BMSC-hydrogel, in which GAG and total collagen contents reached to ∼61% and 43% of native levels, respectively (Fig. S8). Importantly, *in vivo* results further demonstrated that BMSC-loaded **photo-dECM** hydrogel could successfully regenerate cartilage-like tissue in the subcutaneous environment without any extra chondrogenic factors 4 weeks after implantation (Fig. S9), providing the most direct evidence that the **photo-dECM** hydrogel had robust regulatory function for chondrogenic differentiation and cartilage regeneration of BMSCs. Proteomic analysis further confirmed the existence of abundant cartilage-specific proteins, growth factors, and corresponding pathway factors (Fig. 1E and S10), which provided a reasonable explanation for the chondrogenic mechanism of the **photo-dECM** hydrogel.

### 3.3 In vivo engineered bone regeneration based on endochondral ossification

To mimic the physiological developmental process of subchondral bone, the feasibility of engineered bone regeneration based on endochondral ossification strategy was investigated by creating a biomimetic osteogenic microenvironment. The design concept of dynamic regulation for endochondral ossification composed a biomimetic chondrogenic microenvironment mediated by **photo-dECM**_**C**_**/Col** hydrogel (chondrogenic regulation confirmed as described above) and time-dependent osteogenic regulation mediated by decalcified bone matrix.

**Fig. 3:**
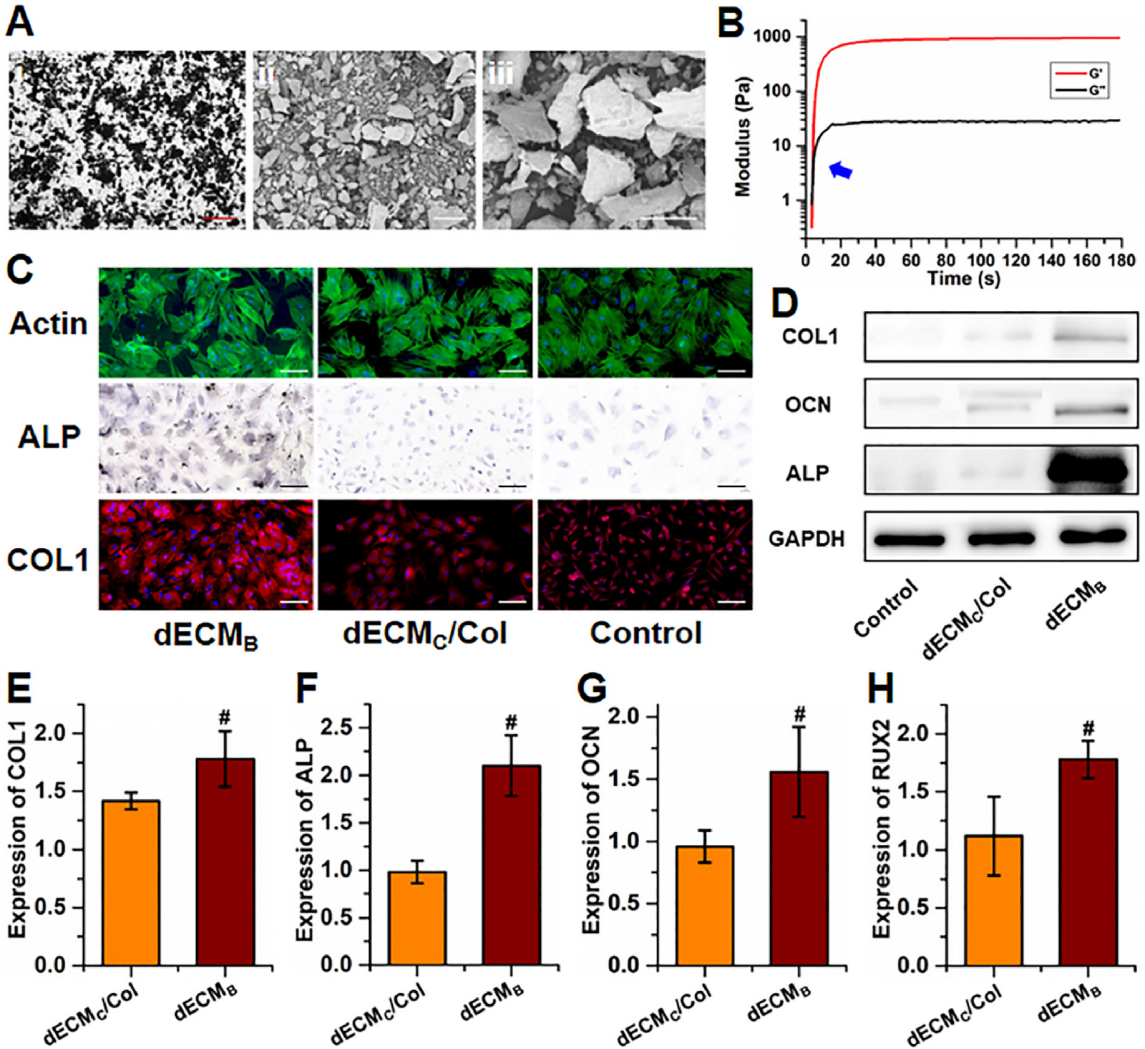
*In vitro* osteogenesis evaluation of decalcified bone matrix. (A) Light microscopy graph (i) and scanning electron microscopy images (ii, iii) of decalcified bone matrix. Red scale bar ​= ​500 ​μm; white scale bar ​= ​200 ​μm. (B) Rheology analysis of **photo-dECM**_**C**_**/Col/dECM**_**B**_ hydrogel. (C) Osteogenic staining of F-actin, ALP, and COL1 of BMSCs co-cultured with or without gel ingredients (**dECM**_**B**_, and **dECM**_**C**_/**Col**) at day 14. (D) Cell lysates were harvested and analyzed by western blot to detect COL1, OCN, ALP and GAPDH. (E–H) Expression of osteogenic genes (*COL1*, *ALP*, *OCN*, and *RUNX2*) in BMSCs cultured with gel ingredients (**dECM**_**B**_, and **dECM**_**C**_/**Col**) at day 14 (#p ​< ​0.05, compared with the control group).

Decalcified bone matrix was obtained from bone tissue, namely bone-derived dECM (**dECM**_**B**_), whose strong osteogenic activity has been reported previously [38]. Micrograph and scanning electron micrograph (SEM) results showed a uniform micron-sized powder of **dECM**_**B**_ (∼100 ​μm; Fig. 3A). After the addition of **dECM**_**B**_ powder into **photo-dECM**_**C**_**/Col** hydrogel, the rheological test demonstrated that the combination of **dECM**_**B**_ powder and **photo-dECM**_**C**_**/Col** hydrogel still showed satisfactory gelling properties with rapid gel formation (∼5 ​s) and slightly enhanced elastic modulus (∼1000 ​Pa; Fig. 3B).

The osteogenic capability of **dECM**_**B**_ was further evaluated by co-culturing BMSCs with **dECM**_**B**_ powder (0.5% w/v). The **dECM**_**B**_-treated group showed obvious bone-specific staining of COL1 and ALP, whereas the **dECM**_**C**_**/Col** and non-treated (control) groups exhibited relatively weak staining (Fig. 3C). Expression of osteogenic protein biomarkers (COL1, OCN, ALP) and bone-related gene expression (*COL1*, *ALP*, *OCN*, and *RUNX2*) in the **dECM**_**B**_-treated group were higher than in the **dECM**_**C**_**/Col** or control groups, and displayed a significant upregulation at 14 days under **dECM**_**B**_ osteogenic stimulation (Fig. 3D–H). This indicated that **dECM**_**B**_ could provide a strong bone inductive microenvironment to promote osteogenic differentiation of BMSCs. Proteomic analysis further confirmed the existence of abundant bone-specific proteins, growth factors, and corresponding pathway factors (Fig. S10), which provided a reasonable explanation for the osteogenic mechanism of **dECM**_**B**_ particles.

**Fig. 4:**
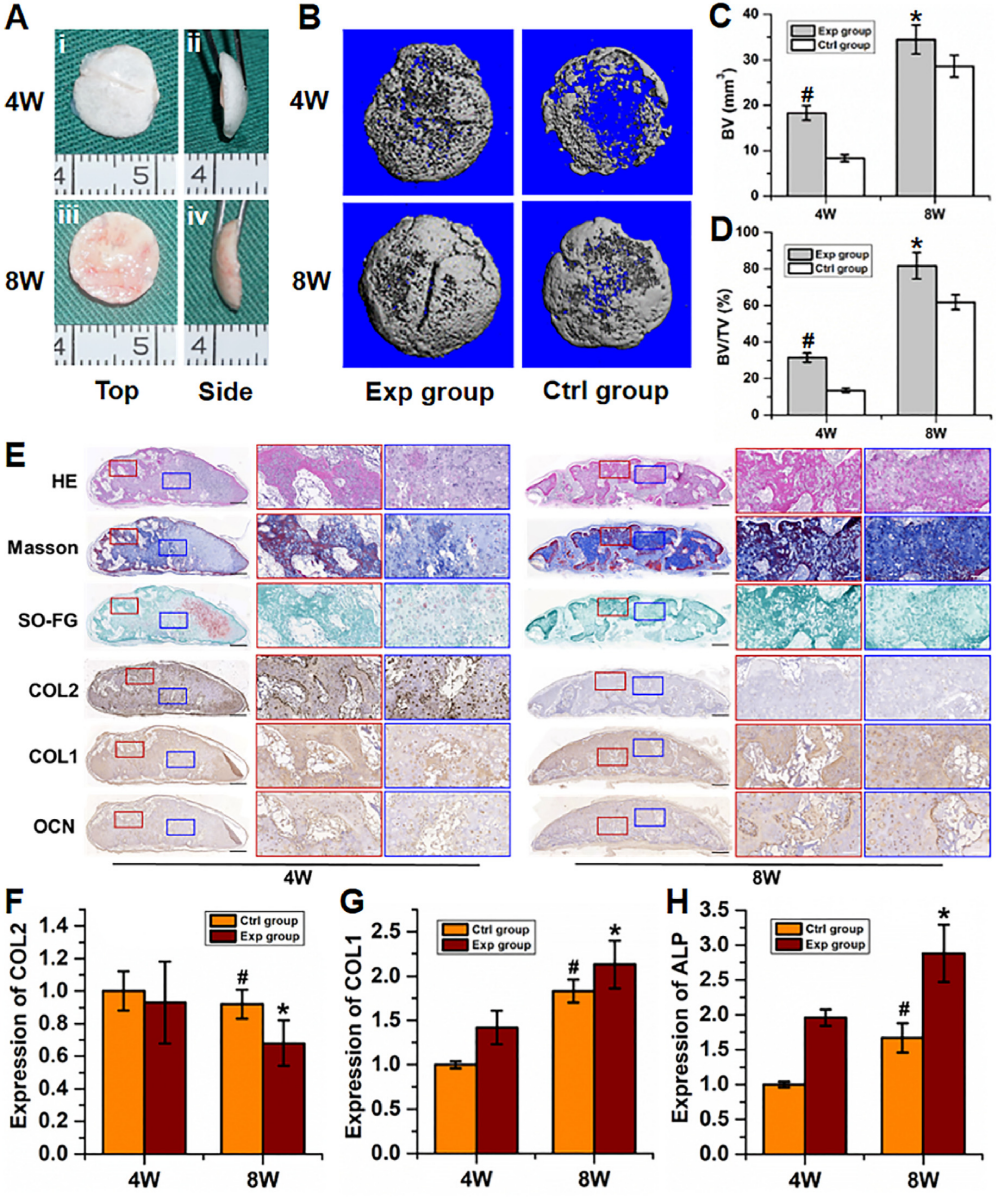
*In vivo* engineered bone regeneration of endochondral ossification. (A) Gross view of the regenerated bone at 4 and 8 weeks after implantation. (B) Representative images of Micro-CT reconstructions of Exp and Ctrl groups at 4 and 8 weeks after implantation. (C, D) Quantitative analysis of morphometric indices, including the bone volume (BV; C) and bone volume fraction (BV/TV; D) of the regenerated bone tissue. (E) Representative histological images of H&E, Masson, Safranin-O/fast green, type Ⅱ, I collagen and OCN staining of the regenerated bone in the Exp group at 4 and 8 weeks. Black scale bar ​= ​500 ​μm; white scale bar ​= ​200 ​μm. (F–H) Gene expression (*COL2*, *COL1*, and *ALP*) of the regenerated bone at 4 and 8 weeks. (#p, ∗p ​< ​0.01, compared with the same group at 4 weeks).

Furthermore, BMSC-loaded **photo-dECM**_**C**_**/Col/dECM**_**B**_ hydrogels (10% w/v, **dECM**_**C**_**-MA**:**ColMA** ​= ​1:1, 5% w/v **dECM**_**B**_, “Exp group”) were subcutaneously implanted into nude mice to evaluate the feasibility of engineered bone regeneration based on endochondral ossification strategy; BMSC-loaded **photo-dECM**_**C**_**/Col** hydrogels (10% w/v, **dECM**_**C**_**-MA**:**ColMA** ​= ​1:1, “Ctrl group”) were used as the control. The *in vivo* results demonstrated that BMSC-loaded **photo-dECM**_**C**_**/Col/dECM**_**B**_ hydrogels successfully regenerated new bone tissue in the subcutaneous environment, which exhibited a gradual mature tendency from ivory white cartilage-like appearance at 4 weeks to reddish bone-like appearance at 8 weeks (Fig. 4A). Micro-CT showed that mature bone regeneration was observed as early as 4 weeks at both central and marginal areas in the Exp group, which was further enhanced at 8 weeks. In the Ctrl group, however, mature bone regeneration at 4 weeks was primarily observed in marginal areas (which is highly related to the influence of subcutaneous vascularized environment), but not in central areas (Fig. 4B). Quantitative analysis data demonstrated that both bone volume (BV) and ratio of bone volume to tissue volume (BV/TV) in the Exp group were significantly higher than those in the Ctrl group at both 4 and 8 weeks, indicating that the existence of **dECM**_**B**_ could effectively boost bone regeneration (Fig. 4C and D).

Histological examinations further supported the above gross observation and micro-CT results. As shown in Fig. 4E and S9, positive safranin-O and type II collagen staining assays with typical lacuna structures were observed in both the Exp and Ctrl groups at 4 weeks, indicating that they exhibited cartilage regeneration in the early stage. Differently, bone-specific staining (Masson, fast green, type I collagen and OCN staining) demonstrated that bone-specific matrix deposition was observed at both central and marginal areas in the Exp group, while bone-specific matrix at 4 weeks was mainly observed at marginal areas, but not at central areas. These results indicated that the existence of **dECM**_**B**_ in the Exp group could initiate bone regeneration of endochondral ossification earlier than the Ctrl group. At 8 weeks, the samples in the Exp group exhibited relatively homogeneous and mature bone-like tissue at both central and marginal areas with stronger bone-specific matrix staining and weaker cartilage-specific matrix staining compared to 4 weeks, indicating a time-dependent tendency of endochondral ossification. Consistent with histological examinations, gene expression analysis further confirmed that osteogenic genes (*ALP* and *COL1*) in the Exp group were significantly upregulated while the chondrogenic gene (*COL2*) was significantly downregulated over the time since implantation (Fig. 4F–H). In the Ctrl group, although the samples at 8 weeks presented stronger bone regeneration at marginal areas compared to 4 weeks (due to the influence of subcutaneous vascularized environment), the whole samples mainly presented cartilage-like tissue and relatively homogeneous cartilage-like tissue was still observed at central areas, indicating that the chondrogenic microenvironment created by **photo-dECM**_**C**_**/Col** hydrogel provided strong regulation on cartilage regeneration for BMSCs.

All of the above results demonstrated that the **photo-dECM**_**C**_**/Col/dECM**_**B**_ hydrogel scaffold could achieve dynamic regulation of bone regeneration through time-dependent endochondral ossification mediated by both chondrogenic and osteogenic microenvironments.

### 3.4 In vivo biphasic cartilage-bone integrated tissue regeneration

**Fig. 5:**
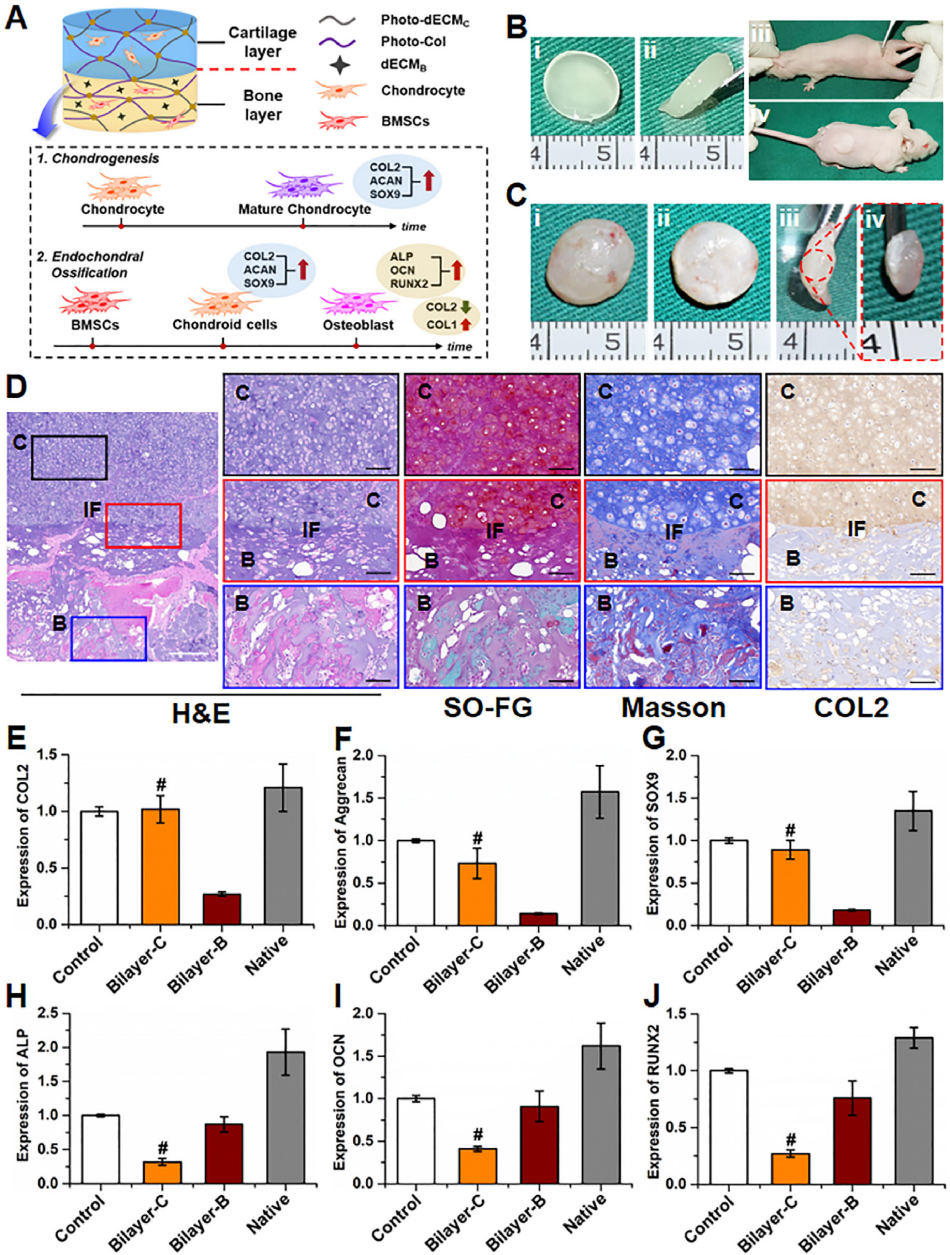
*In vivo* biphasic cartilage-bone integrated tissue regeneration. (A) Schematic illustration of the bilayered construct design (upper: cartilage layer; bottom: bone layer), and the pathway of chondrogenesis and endochondral ossification. (B) Culture procedures for cartilage-bone construct subcutaneously implanted in nude mice. (C) Gross view of the regenerated cartilage-bone integrated tissue at 8 weeks after implantation. (D) Representative histological images of H&E, Safranin-O/fast green, Masson, and type Ⅱ collagen staining of the regenerated cartilage-bone integrated tissue at 8 weeks after implantation. C: cartilage layer; B: bone layer; IF: cartilage-bone interface. White scale bar ​= ​500 ​μm; Black scale bar ​= ​200 ​μm. (E–J) Expression of cartilage-related genes (*COL2*, *AGG*, and *SOX9*) and bone-related genes (*ALP*, *OCN*, and *RUNX2*) of the regenerated cartilage-bone integrated tissue at 8 weeks. (#p ​< ​0.01, compared with the corresponding bone layer). Control group represents the corresponding tissue-engineered cartilage or bone.

The feasibility of *in vivo* biphasic cartilage-bone integrated tissue regeneration was next investigated. To assure stable chondrogenesis, chondrocytes were chosen as the seed cell source for cartilage regeneration in biphasic integrated tissue, while for bone regeneration, we continued to use the BMSC-loaded **photo-dECM**_**C**_**/Col/dECM**_**B**_ hydrogel for dynamic regulation of endochondral ossification. We found that sequential combination of BMSC-loaded **photo-dECM**_**C**_**/Col/dECM**_**B**_ hydrogel and chondrocyte-loaded **photo-dECM**_**C**_**/Col** hydrogel successfully regenerated cartilage-bone integrated tissue with seamless interfacial integration (Fig. 5A–C). Histological examinations further confirmed biphasic integrated tissue regeneration with seamless interfacial integration and tissue-specific regeneration, showing cartilage-specific staining in the cartilage layer and bone-specific staining in the bone layer 8 weeks after *in vivo* implantation (Fig. 5D and S11). qPCR also validated that cartilage-related genes (*COL2*, *AGG*, and *SOX9*) were expressed at significantly higher levels in the cartilage layer than the bone layer, while bone-related genes (*ALP*, *OCN*, and *RUNX2*) were expressed at higher levels in the bone layer than in the cartilage layer at 8 weeks. Overall, this indicates that satisfactory biphasic structure of hybrid cartilage-bone tissue was successfully fabricated (Fig. 5E–J).

### 3.5 Osteochondral repair based on photo-crosslinkable dECM hydrogels

**Fig. 6:**
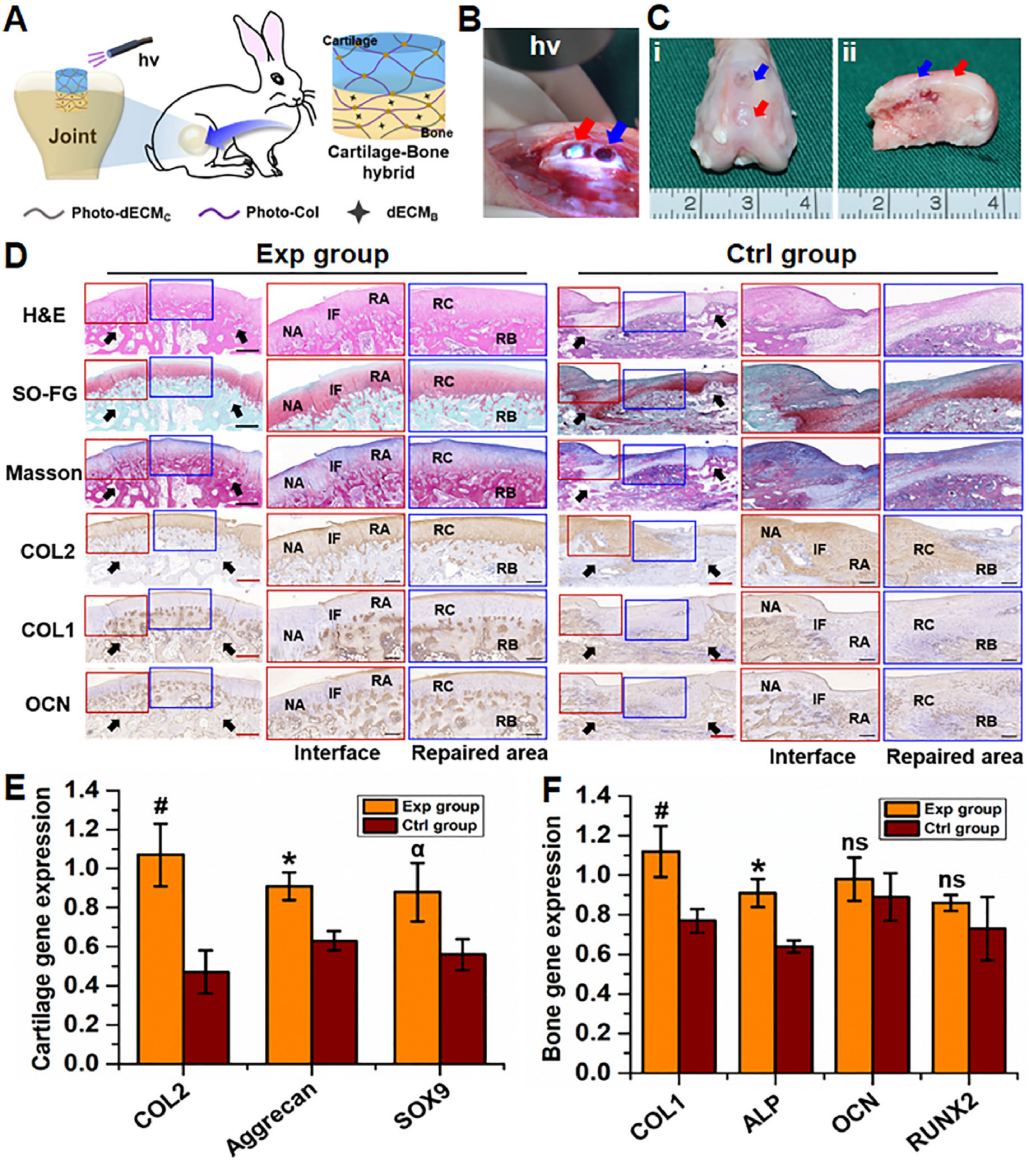
Osteochondral repair based on the biomimetic photo-crosslinkable dECM hydrogels. (A) Schematic illustration of the repair of osteochondral defects based on the biomimetic cartilage-bone integrated scaffolds. (B) Surgical procedures of *in situ* filling osteochondral defects using **photo-dECM** hydrogel upon light irradiation (365 ​nm LED, 20 ​mW/cm^2^). (C) Macroscopic images of the osteochondral joints from Exp and Ctrl groups at 12 weeks after surgery. Red arrow: Exp group; Blue arrow: Ctrl group. (D) Representative histological images of H&E, Safranin-O/fast green, Masson, type Ⅱ, I collagen and OCN staining of osteochondral defect area of Exp and Ctrl groups at 12 weeks after surgery. RA: repaired area; IF: interface; NA: native area; RC: repaired cartilage; RB: repaired bone. Black arrows indicate repaired regions. Black scale bar ​= ​1 ​mm; white scale bar ​= ​200 ​μm. (E, F) Expression of cartilage-related genes (*COL2*, *AGG*, and *SOX9*; E) and bone-related genes (*COL1*, *ALP*, *OCN*, and *RUNX2*; F) of the regenerated osteochondral tissue at 12 weeks. (#p, ∗p, ^α^p ​< ​0.05, compared with the Ctrl group, ns represented no significant difference).

To evaluate the feasibility of osteochondral defect repair using the current biphasic cartilage-bone integrated scaffolds, standard osteochondral defects with cylindrical shape (4 ​mm in diameter and depth) in a rabbit model were created and sequentially repaired with cell-free bone phasic scaffold (**photo-dECM**_**C**_**/Col/dECM**_**B**_ hydrogel, implanted into bone defect region at 3 ​mm depth), followed by cell-free cartilage phasic scaffold (**photo-dECM**_**C**_**/Col** hydrogel, implanted into the cartilage defect region at 1 ​mm depth; Fig. 6A). As shown in Fig. 6B, the bilayered hydrogel scaffold could rapidly gel in the defect region upon light irradiation, integrate with the surrounding tissue, and fill in the defects with a smooth surface (the Exp group). After 12 weeks, gross observation showed that the osteochondral defects in the Exp group were satisfactorily repaired with regenerated cartilage-like tissue at the cartilage layer and regenerated bone-like tissue at the bone layer with favorable interfacial healing (Fig. 6C). Histological evaluation further confirmed that the cartilage region of osteochondral defects in the Exp group were successfully repaired by mature hyaline cartilage with typical lacuna structures, cartilage-specific matrix deposition, and negative staining of COL1 and OCN (Fig. 6D and Fig. S12). Meanwhile, the bone region of osteochondral defects in the Exp group were successfully repaired by mature cancellous bone with typical trabecula structures, bone-specific matrix deposition, and negative staining of COL2. Importantly, both the regenerated cartilage and subchondral bone could integrate well with the host tissues. Moreover, the epiphyseal line-like structure close to surrounding native cartilage could be observed between regenerated cartilage and bone. Consistent with prior reports, in non-treated defects (the Ctrl group), both gross and histological examinations demonstrated that the osteochondral defects had unsatisfactory repair in both the cartilage and bone regions. Gene expression analysis further confirmed that the expression levels of both chondrogenic genes (*COL2*, *AGG*, and *SOX9*) and osteogenic genes (*COL1*, *ALP*, *OCN* and *RUNX2*) were significantly higher in the Exp group than in the Ctrl group (Fig. 6E and F). The above results definitively demonstrated that *in situ* sequential implantation of the cell-free biphasic biomimetic scaffolds could successfully achieve satisfactory tissue-specific repair of osteochondral defects with regenerated cartilage and subchondral bone. We hypothesize that this repair was mainly due to the activation of endogenous repair mediated by tissue-specific differentiation of homing BMSCs, which was regulated by the biphasic cartilage-bone integrated scaffolds.

## 4 Discussion

To date, there have been no significant breakthroughs in biphasic cartilage-bone integrated regeneration and *in situ* defect repair due to a lack of ideal biomimetic scaffolds. Photo-crosslinkable hydrogels, based on tissue-specific dECM, have been seen as one of the most promising strategies to fabricate biomimetic scaffolds. However, the biphasic integrated scaffolds with accurate cartilage and bone biomimetic microenvironments and dynamic regulation of endochondral ossification have not yet been developed. Here, we developed a biphasic cartilage-bone integrated scaffold based on photo-crosslinkable cartilage/bone-derived dECM, which was able to achieve biomimic and reconstruction of the biphasic cartilage-bone integrated microenvironment. The cartilage biomimetic scaffolds based on **photo-dECM**_**C**_**/Col** hydrogels successfully regulated chondrogenesis of BMSCs, while the bone biomimetic scaffolds based on **photo-dECM**_**C**_**/Col/dECM**_**B**_ hydrogels efficiently regulated osteogenesis of BMSCs by time-dependent endochondral ossification. More importantly, the biphasic cartilage-bone integrated tissue was successfully regenerated with seamless interfacial integration based on the biphasic biomimetic scaffolds. Articular osteochondral defects were satisfactorily repaired *in situ*, with regenerated cartilage and subchondral bone based on the cell-free biphasic biomimetic scaffolds. Thus, the current study provides a novel strategy and ideal biomimetic scaffold for biphasic cartilage-bone integrated regeneration and *in situ* defect repair.

Preparing cartilage- and bone-specific photo-crosslinkable hydrogels with tissue-specific biomimetic ingredients and satisfactory gelling properties is the first challenge that we faced. The main challenge is the difficulty to create decellularized and water-soluble treatment because of the compact structure of cartilage and highly mineralized components of bone. In fact, by the complex treatments of physical, chemical, or enzymatic approaches, the cartilage tissue could be prepared into soluble dECM hydrogels. However, these complex treatments will inevitably cause partial loss of tissue-specific biomemitic ingredients and poor gelling properties, which is the main bottleneck for preparing photo-crosslinkable cartilage dECM hydrogels. Recently, Visscher et al. first reported that cartilage-derived dECM could be successfully processed into a photo-crosslinkable hydrogel by pepsin digestion, methacrylation treatment, and adding of **GelMA** [37]. This study demonstrated the feasibility of *in vitro* cartilage regeneration using chondrocyte-loaded hydrogel, but failed to provide related evidence about the chondrogenic regulation of BMSCs. In the concurrent study, we adopted the collagenase digestion strategy as a water-soluble treatment to decrease the negative effect of type Ⅱ collagen triggering inflammation [50,51]. Additionally, **ColMA** derived from skin was further added to counteract the dramatic loss of collagen components caused by collagenase digestion (Fig. 1D). We found that adding **ColMA** significantly enhanced the gelling properties, implying that the removal of collagen components by collagenase digestion was the major reason for poor gelling. According to the proteomic analysis, most cartilage-related bioactive components could be detected in **photo-dECM**_**C**_ hydrogels, indicating efficient biomimicking of the cartilage-specific microenvironment.

Compared to cartilage dECM hydrogels, the preparation of dECM hydrogels based on bone tissue was a greater challenge. Even after complex treatments by decellularization and enzymic digestion, the bone-derived dECM hydrogels still could not be fabricated. In fact, no water-soluble bone-related bioactive ingredients were obtained after the complex treatments in most instances. However, the specific structure and bioactive ingredients of bone matrix are known to be important in achieving a biomimetic bone microenvironment. To prepare a photo-crosslinkable hydrogel system with bone biomimetic function, we proposed a hybrid approach combining **photo-dECM**_**C**_**/Col** hydrogel and the powder-like **dECM**_**B**_. The **photo-dECM**_**C**_**/Col** hydrogel provided a chondrogenic microenvironment, which promoted the chondrogenic differentiation and cartilage regeneration by BMSCs. The **dECM**_**B**_ particles provided an osteogenic microenvironment with the function of both bone conduction and induction, which further regulated the ossification of BMSC regenerated cartilage in the **photo-dECM**_**C**_**/Col/dECM**_**B**_ system. Thus, the novel design in combining **photo-dECM**_**C**_**/Col** hydrogel and **dECM**_**B**_ provided the potential for time-dependent endochondral ossification regulation based on BMSCs. The current results demonstrated that most bone-related bioactive components and structures were retained in the **dECM**_**B**_ particles. More importantly, the mixture of **dECM**_**B**_ particles and **photo-dECM**_**C**_**/Col** hydrogel still showed satisfactory gelling properties suitable for cell loading.

Whether the photo-crosslinkable biomimetic dECM hydrogel system has the function of regulating cartilage and bone regeneration of BMSCs is an important issue. In cartilage regulation, the current results confirmed that the **photo-dECM**_**C**_**/Col** hydrogel showed favorable biocompatibility for supporting cell survival and proliferation, effectively promoted chondrogenic differentiation of BMSCs, and efficiently regulated cartilage regeneration of BMSCs *in vitro*. More importantly, BMSC-loaded **photo-dECM**_**C**_**/Col** hydrogel successfully regenerated cartilage tissue in the subcutaneous environment without any other chondrogenic factors. Therefore, both *in vitro* and *in vivo* results indicated that the **photo-dECM**_**C**_**/Col** hydrogel had robust regulatory function for chondrogenic differentiation and cartilage regeneration of BMSCs, which was apparently attributed to the biomimetic chondrogenic microenvironment provided by cartilage-specific bioactive ingredients in the hydrogel system. In bone regulation, *in vitro* experiments demonstrated that **dECM**_**B**_ particles effectively provided an osteogenic biomimetic microenvironment, as they promoted osteogenic differentiation of BMSCs. Notably, *in vivo* experiments demonstrated that BMSC-loaded **photo-dECM**_**C**_**/Col/dECM**_**B**_ hydrogel regenerated cartilage-like tissue in the early stage, and gradually presented time-dependent ossified tendencies. These results implied that the hydrogel system could achieve sequential regulation for endochondral ossification of BMSCs, which we ascribed to the novel design in both chondrogenic and osteogenic microenvironments.

Because both engineered cartilage and bone could be successfully regenerated separately, whether the biphasic cartilage-bone integrated tissue might be constructed becomes another concerned issue. Ectopic chondrogenesis of BMCSs in the subcutaneous environment is known to show difficulty in maintaining a stable cartilage phenotype. To assure stable chondrogenesis in an ectopic non-chondrogenic microenvironment, chondrocytes were chosen as seed cell source to allow for cartilage regeneration in biphasic integrated tissue. For bone regeneration in biphasic integrated tissue, we continued to use the BMSC-loaded **photo-dECM**_**C**_**/Col/dECM**_**B**_ hydrogel for dynamic regulation of endochondral ossification. As expected, the sequential combination of BMSC-loaded **photo-dECM**_**C**_**/Col/dECM**_**B**_ hydrogel and chondrocyte-loaded **photo-dECM**_**C**_**/Col** hydrogel successfully regenerated cartilage-bone integrated tissue with seamless interfacial integration. However, despite the satisfactory interface integration, it failed to form the epiphyseal line structure, which may be related to a lack of mechanical stimulation in the articular environment that is important for remolding regenerated tissue.

Finally, whether the cell-free biphasic biomimetic scaffolds could successfully repair articular osteochondral defects *in situ* was a very important preclinical consideration. We here demonstrated that the *in situ* sequential implantation of **photo-dECM**_**C**_**/Col/dECM**_**B**_ hydrogel (bone defect region) and **photo-dECM**_**C**_**/Col** hydrogel (cartilage defect region) successfully achieved satisfactory tissue-specific repair of osteochondral defects, with regenerated cartilage in the cartilage defect region and subchondral bone in the bone defect region. We hypothesize that the satisfactory tissue-specific repair can be attributed to the activation of endogenous repair mediated by tissue-specific differentiation of autogenous homing BMSCs, which is accurately regulated *in situ* by the cartilage-bone biphasic biomimetic scaffolds. In addition, both regenerated cartilage and subchondral bone presented satisfactory interfacial integration with the surrounding native tissue. The regenerated cartilage achieved normal thickness with the epiphyseal line structure close to surrounding native cartilage, which could be related to the mechanical stimulation of the articular environment that efficiently regulated regenerated tissue remolding. Indeed, it is also important to perform longer term animal studies in the future to confirm the long-term phenotypic stability of repaired tissue.

## 5 Conclusion

In summary, the current study developed a novel photo-crosslinkable hydrogel system based on cartilage/bone-derived dECM to achieve biomimic and reconstruction of biphasic cartilage-bone integrated microenvironment. The cartilage biomimetic scaffolds successfully regulated chondrogenesis of BMSCs, while the bone biomimetic scaffolds efficiently regulated osteogenesis of BMSCs by time-dependent endochondral ossification. Furthermore, the biphasic cartilage-bone integrated tissue could be successfully regenerated with seamless interfacial integration based on the biphasic biomimetic scaffolds. Eventually, sequential implantation of the cell-free biphasic biomimetic scaffolds *in situ* successfully achieved satisfactory tissue-specific repair of osteochondral defects with regenerated cartilage and subchondral bone. Further preclinical studies are needed to evaluate factors, such as biosafety and the feasiblility of repairing large-scale osteochondral defects in larger animals. Nonetheless, the current study provided a novel strategy and identified an ideal biomimetic scaffold for cartilage-bone integrated regeneration and *in situ* defect repair.

## Credit author statement

Y. H., Y.H., B. B., J. H., W. R., X. W., and G. Z. designed the experiments. Y. H., Y.H., B. B., J. H., G. H., Z. C., X. W., and M. Y. performed the experiments. Y. H., Y.H., B. B., J. H., X. W., H. C., and Y. Z. analyzed the data. Y. H., and G. Z. wrote the manuscript.

## Declaration of competing interest

The authors declare that they have no known competing financial interests or personal relationships that could have appeared to influence the work reported in this paper.

## Data Availability

The data that has been used is confidential.
