# Supplementary material for: Fabrication of biphasic cartilage-bone integrated scaffolds based on tissue-specific photo-crosslinkable acellular matrix hydrogels
Source: Mater Today Bio. 2022 Nov 8;17:100489. doi: 10.1016/j.mtbio.2022.100489 (PMC9663535; doi:10.1016/j.mtbio.2022.100489)
Supplement: Multimedia component 1 [file mmc1.docx]

Supporting Information

**Fabrication of biphasic cartilage-bone integrated scaffolds based on tissue-specific photo-crosslinkable acellular matrix hydrogels**

Yujie Hua^a,d,e‡^, Yingying Huo^a,d‡^, Baoshuai Bai^f‡^, Junxiang Hao^c,d‡^, Guanhuai Hu^e^, Zheng Ci^c,d^, Xiaodi Wu^c,d^, Mengyuan Yu^e^, Xin Wang^g^, Hong Chen^g^, Wenjie Ren^e^*, Yixin Zhang^a^, Xiaoyun Wang^b^*, and Guangdong Zhou^a,c,d,e^*

^a^Department of Plastic and Reconstructive Surgery, Shanghai Ninth People’s Hospital, Shanghai Jiao Tong University School of Medicine, Shanghai Key Laboratory of Tissue Engineering, Shanghai, PR China.

^b^Department of Plastic Surgery, Tongren Hospital, Shanghai Jiao Tong University School of Medicine, Shanghai Key Laboratory of Tissue Engineering, Shanghai, PR China.

^c^Research Institute of Plastic Surgery, Weifang Medical University, Weifang, Shandong, PR China.

^d^National Tissue Engineering Center of China, Shanghai, PR China.

^e^Institute of Regenerative Medicine and Orthopedics, Institutes of Health Central Plain, Xinxiang Medical University, Xinxiang, Henan, PR China.

^f^Department of Orthopaedics, Qilu Hospital of Shangdong University Centre for Orthopaedics, Advanced Medical Research Institute, Shangdong University, Shangdong, PR China.

^g^Department of Hand Surgery, Ningbo Sixth Hospital, Zhejiang, PR China.

^‡^These authors contributed equally: Yujie Hua, Yingying Huo, Baoshuai Bai, Junxiang Hao.
***Corresponding author. E-mail: [wjren1966@163.com](mailto:wjren1966@163.com); [gaokongliuyun@126.com](mailto:gaokongliuyun@126.com); [guangdongzhou@126.com](mailto:guangdongzhou@126.com)


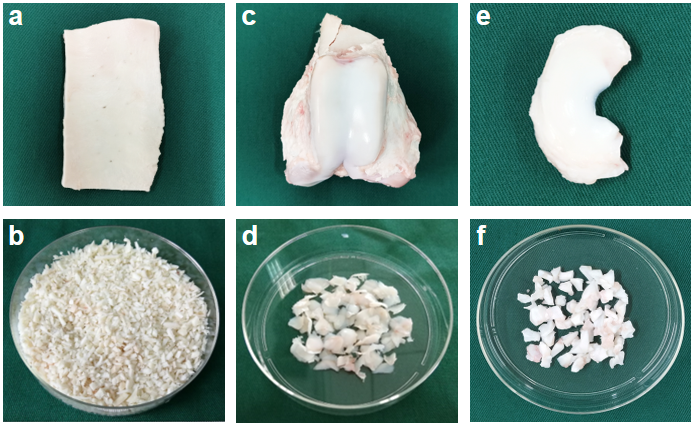


**Fig. S1.** Photographs of cartilage tissue obtained from porcine ear (a, b), joint (c, d), and meniscus (e, f).

**
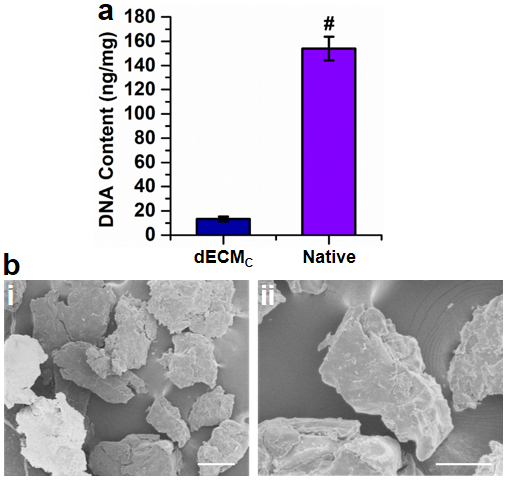
**

**Fig. S2.** a) DNA content before and after decellularization (^#^p < 0.05, compared with the native tissue). b) Scanning electron microscopy images of **dECM_C_**. Red scale bar = 500 μm; White scale bar = 200 μm.

**
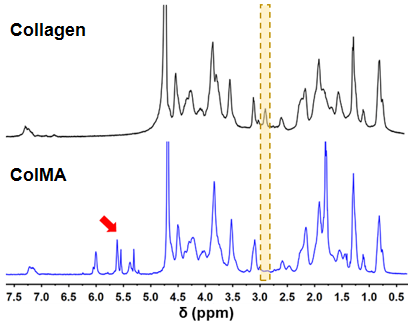
**

**Fig. S3.** ^1^H NMR spectra of collagen and **ColMA** polymers. The signal peaks of 5.2-6.2 ppm indicated the successful graft of methacrylate groups. The substitution degree was determined by the integral ratio of the proton peaks at 5.2-6.2 ppm to the peak at 2.8 ppm (amino groups of collagen), indicating 91% of methacrylate-modified collagen (**ColMA**).


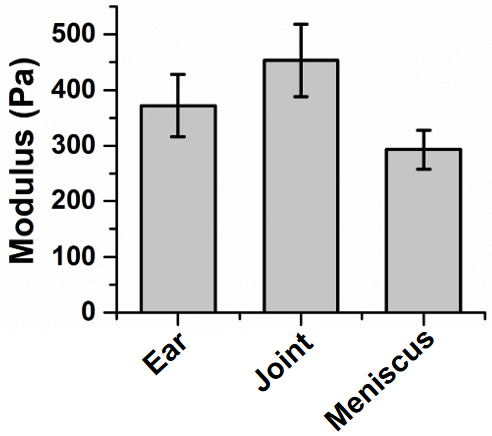


**Fig. S4.** Comparison of elastic modulus of 10% w/v **photo-dECM_C_** hydrogel derived from different source of cartilage tissue.





**Fig. S5.** The cell viability of the gel precursor of **photo-dECM** hydrogels on the chondrocytes was evaluated by CCK-8 assay (n = 4).


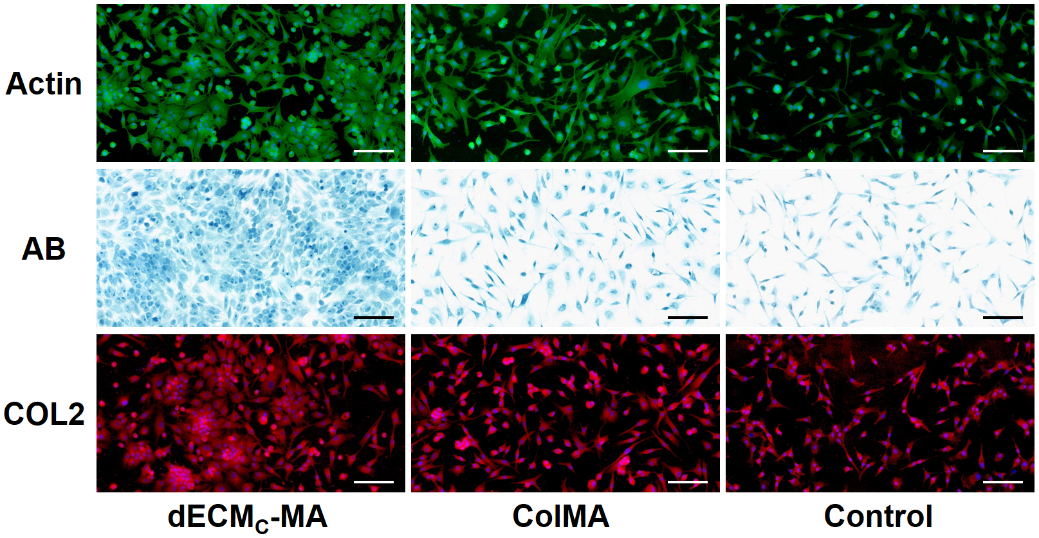


**Fig. S6.** Chondrogenic staining of F-actin, Alcian Blue (AB), and COL2 of chondrocytes co-cultured with or without gel precursor (**dECM_C_-MA** and **ColMA**) at day 14.


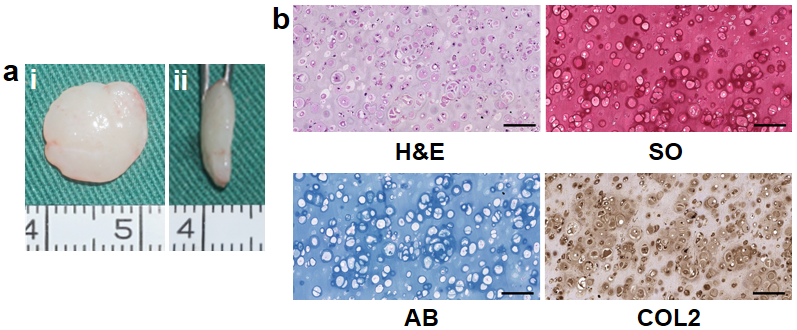


**Fig. S7.** a) Gross view of the regenerated cartilage at 8 weeks *in vivo* (i, ii). b) Representative histological images of H&E, Safranin-O, Alcian Blue, and type Ⅱ collagen staining of the regenerated cartilage at 8 weeks *in vivo*. Black scale bar = 200 μm.


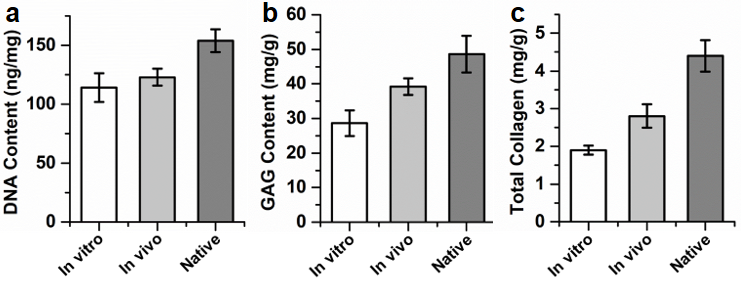


**Fig. S8.** Quantitative evaluations of DNA (a), GAG contents (b), and total collagen (c) of the regenerated cartilage at 8 weeks *in vitro* and *in vivo*.


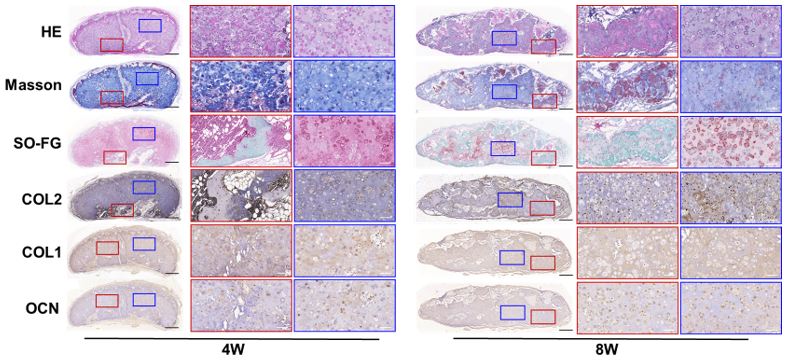


**Fig. S9.** Representative histological images of H&E, Masson, Safranin-O/fast green, type Ⅱ, Ⅰ collagen and OCN of the regenerated bone in the Ctrl group at 4 and 8 weeks. Black scale bar = 500 μm; White scale bar = 200 μm.


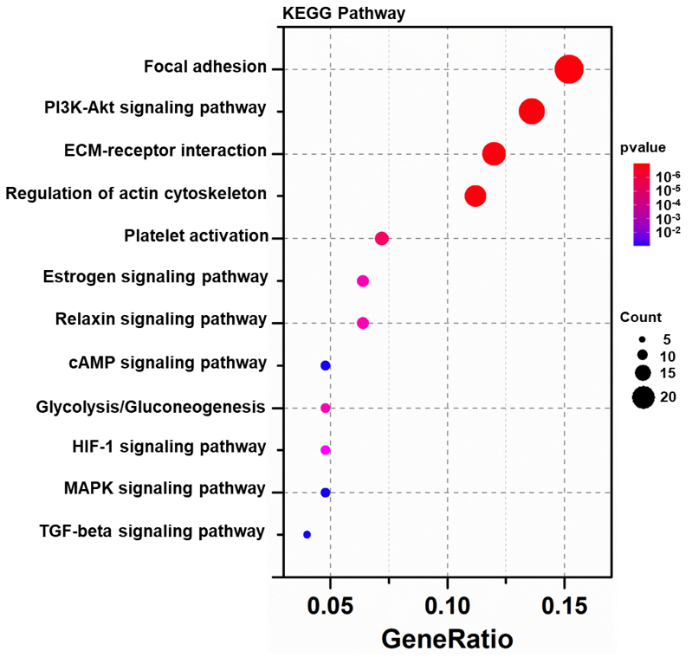


**Fig. S10.** The statistical analysis of KEEG pathway of **dECM_B_**.

**The detected proteins components of dECM_C_:**

F1SN67; I3LUR7; F1RQI0; A0A5G2QQE9; A0A5G2QM06; F1SFA7; A0A287ATP0; A0A287B863; A0A5G2RKJ7; I3LPA7; A0A286ZT13; F1S0V3; F1REZ1; A0A287A461; F1RYI8; K7GKY0; F1RMJ4; A0A287AE16; F1SS26; A0A5G2R860; A0A5G2RD69; A0A288CFV5; F1S2E2; Q6S4N2; A0A286ZXU2; F1S9Q3; F1RPH0; Q06AS6; A0A5G2RI50; F1SAX3; A0A287A5B4; F1SPZ5; A0A286ZWS8; P00761; A0A480WPU1; A0A287AK19; A0A287AEL2; F1SCU3; P00355; A0A287A217; P02543; F1S6B5; A0A5G2QSK4; A0A287BIL8; F1SGG6; A0A287AKJ2; I3LDS3; A0A5G2QSR7; A0A288CG57; Q5S1S4; F1RF28; A0A287AZP0; A0A287A7Q3; F1SGG3; F1SQL2; P02067; A0A5G2QPS5; I3LII3; A0A5G2QAE8; A0A287BAC5; A0A287ACK8; F2Z4Y1; A0A287BES2; F2Z5U4; F2Z5K2; A0A287AT18; A0A286ZSD4; I3LPT1; P50447; P60662; A0A286ZWI1; A0A5G2QKI1; F1RQR4; A0A5G2QME7; A0A287B4G4; A0A5G2RA57; A0A287BEI1; I3LHI7; F2Z5E2; A0A287APR3; A0A2C9F3E9; F1SAY0; A0A5G2QW05; A0A287AG70; A0A5G2QR34; F1S3P6; F1SII4; A0A287B088; F1SGI7; A0A287A5S7; I3LTB8; A0A287B5A2; A0A286ZX54; A0A287BI18; Q8WNQ7; A0A287B3S3; F1S5H0; A0A5G2R8D7; I3L9V2; F1ST93; A0A287BD64; A0A5G2Q8A7; A0A287A286; A0A480KYX1; A0A5G2REG7; F1RQM2; F1SC20; K9IVK6; A0A287BEC7; F1SUW2; K9IVW4; P08419; I3LDU9; A0A5G2RLP2; Q70KH2; A0A287BGK8; A0A287AMJ5; A0A287BKP9; A0A5G2QYG4; Q8MIA3; A0A5G2QTP2; A0A286ZRB3; I3L554; F1SHN7; B5APU6; I3L7Z6; A0A287BG85; I3LDH3; A0A287A2P1; A0A287ALY1.

**The detected proteins components of dECM_B_:**

F1SN67; I3LUR7; F1RQI0; A0A5G2QQE9; A0A5G2QM06; F1SFA7; A0A287ATP0; A0A287B863; A0A5G2RKJ7; F1SKM1; A0A287AVF2; I3LPA7; A0A286ZT13; F1S0V3; F1REZ1; A0A287A461; A0A5G2R015; F1RYI8; K7GKY0; F1RMJ4; A0A5K1UKC5; F1S6Q3; A0A5G2R860; A0A5G2RD69; A0A288CFV5; Q9GKQ6; F1S2E2; A0A286ZXU2; F1S9Q3; F1RPH0; F1SEN1; Q06AS6; A0A287B583; A0A5G2RI50; F1SAX3; A0A287A5B4; F1SPZ5; A0A286ZWS8; A0A286ZIC1; F1SGJ3; P00761; A0A480WPU1; F1S405; F1S902; A0A287AK19; A0A287AEL2; F1SJB5; P00355; A0A287A217; F1SB42; A0A287ABW3; F1S663; F1SGG6; P29700; I3LID8; A0A287AFC1; I3LD72; A0A287AHS0; A0A5K1U8P1; A5GFU0; F1SV14; I3LDS3; I3LUP6; A0A287BKG2; A0A287AKE4; Q5S1S4; F1RF28; P12069; A0A287AZP0; A0A287A7Q3; P62802; F1S571; I3L7K2; A0A287AYJ8; I3L893; A0A287A7T8; F1SGG3; P12068; A0A480P9F6; A0A287B8I6; P00503; A0A5G2QPS5; I3LII3; K7GKF8; P45845; F1SQ09; A0A287BHY5; F1RGY5; Q9XSD9; A0A5G2QC50; P10668; A0A287BPM1; A0A287A4Y3; P48819; A0A287BES2; F1S021; F6QA08; F2Z5K2; A0A286ZSD4; P50447; A0A5G2QKI1; F1SJ86; I3LB11; I3LQD3; A0A287A2X0; A0A287A2X6; A0A286ZRI5; A0A5G2R299; A0A5G2QM19; A0A5G2QME7; F1RN41; Q6RVA9; A0A5G2RA57; A0A286ZKH3; A0A5G2QRI0; A0A287B0Z8; A0A287BEI1; A0A287A7Y1; A0A287AEJ1; A0A287B854; F1SRC8; A0A287BMP7; A7WLI1; A0A286ZIH7; F1SAY0; A0A5G2QW05; A0A287BEJ3; A0A286ZVB7; F1S3P6; A0A5G2R9D6; F1SGI7; A0A5G2R489; A0A2C9F3F0; F1RN71; A0A5K1UD05; A0A287B5A2; A0A287AP77; P14287; A0A287BI18; A0A5G2QSF4; A0A287A853; A0A5G2QMA3; A0A5G2QX00; A0A5G2QJQ1; P00506; Q8WNQ7; A0A287BQ72; A0A287B3S3; I3L5X3; F1S5H0; B9UJD6; A0A5G2R8D7; F1RSI7; A0A0H5ANC0; A0A5G2R9R4; A0A287A286; A0A5G2REG7; F1RQM2; F1SC20; K9IVK6; F1SE73; A0A140TAK8; A0A287AKV0; F1SUW2; Q9N178; Q8HYY9; Q8SPS7; K9IVW4; A0A5G2QEI5; A0A287BH90; P08419; A0A5G2R920; A0A5G2R543; Q70KH2; F1RZ89; A0A286ZMT0; A0A5G2RBD3; A0A5G2QQB8; K7GM88; A0A5G2R017; A0A5G2QC13; A0A287BKP9; F1SIB1; F2Z594; A0A287BN71; A0A5G2R2K0; A0A5S8KUU6; F1SN69; A0A287AD51; A0A5G2R7N1; A0A5G2QYG4; Q8MIA3; H6UWK6; A0A287B381; B5APU6; A0A5G2QUR1; A0A5G2QMT3; F1RQU7; A0A5G2R2X0; A0A287BG85; A0A287A2P1; A0A287B470; A0A5S6GBZ7.


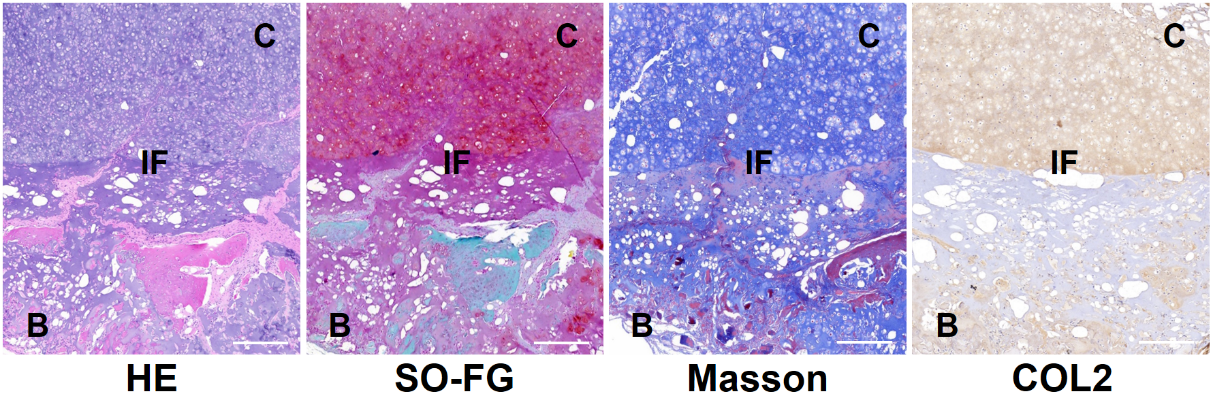


**Fig. S11.** Representative histological images of H&E, Safranin-O/fast green, Masson, and type Ⅱ collagen staining of the regenerated cartilage-bone integrated tissue at 8 weeks after implantation. C: cartilage layer; B: bone layer; IF: cartilage-bone interface. White scale bar = 500 μm.


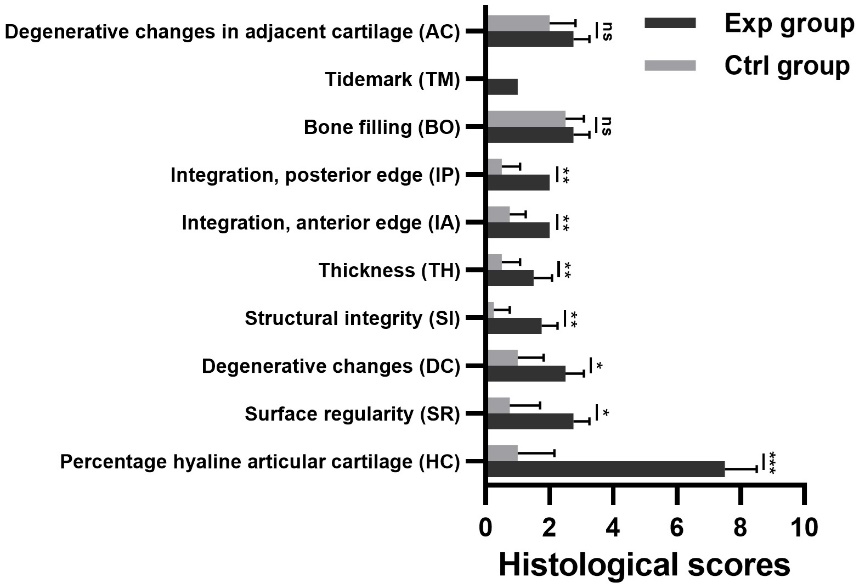


**Fig. S12.** Histological scores of the osteochondral joints from Exp and Ctrl groups at 12 weeks after surgery. (*p < 0.05, **p < 0.01, ***p < 0.001, ns = no significance)
